# Supplementary material for: Identification of photoperiod-induced specific miRNAs in the adrenal glands of Sunite sheep (Ovis aries)
Source: Front Vet Sci. 2022 Jul 22;9:888207. doi: 10.3389/fvets.2022.888207 (PMC9354845; doi:10.3389/fvets.2022.888207)
Supplement: Supplementary file 1 [file Data_Sheet_1.ZIP › Supplementary file/miRDeep2 file.zip!/miRDeep2 file/Know_Structure/SPLP42A2/oar-mir-154b.pdf]

miRBase precursor : oar-mir-154b
Total read count : 2048
oar-miR-154b-5p read count : 1942
oar-miR-154b-3p read count : 102
remaining reads : 4

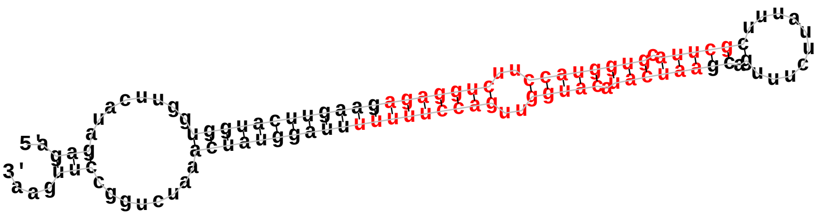

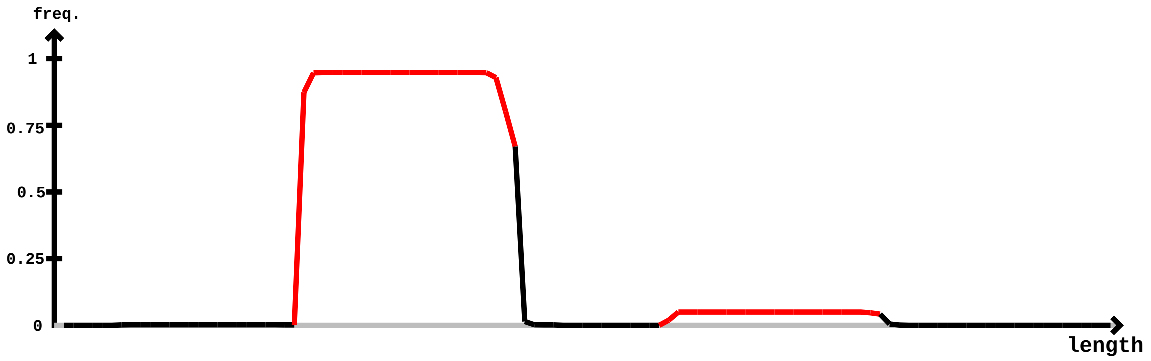

oar-miR-154b-3p

oar-miR-154b-5p

```
5'- agagauacuuggugguacuugaagagaggucuuccauggugcauucgcuuuauucuuugacgaaucauacaugguugaccuuuuuuuagguaucaaaucuggccuugaa   -3'    exp
    .(((........(((((((((((((((((((..((((((((.((((((..........).)))))))).)))))..)))))))))))))))))))........)))...    reads      mm          sample
    .....uacuuggugguacuuga.......................................................................................    1          0           seq
    .....uacuuggugguacuugaag.....................................................................................    2          0           seq
    ......acuuggugguacuugaag.....................................................................................    1          0           seq
    ........................agaggucuuccauggugc...................................................................    1          0           seq
    ........................agaggucuuccauggugca..................................................................    1          0           seq
    ........................agaggucuuccauggugcau.................................................................    37         0           seq
    ........................agaggucuuccauggugcauu................................................................    250        0           seq
    ........................agaggucuuccauggugcauuc...............................................................    259        0           seq
    ........................agaggucuuccauggugcauucg..............................................................    1216       0           seq
    ........................agaggucuuccauggugcauucgc.............................................................    23         0           seq
    ........................agaggucuuccauggugcauucgcu............................................................    1          0           seq
    ........................agaggucuuccauggugcauucgcuuu..........................................................    1          0           seq
    .........................gaggucuuccauggugcau.................................................................    1          0           seq
    .........................gaggucuuccauggugcauu................................................................    9          0           seq
    .........................gaggucuuccauggugcauuc...............................................................    11         0           seq
    .........................gaggucuuccauggugcauucg..............................................................    126        0           seq
    .........................gaggucuuccauggugcauucgc.............................................................    2          0           seq
    .........................gaggucuuccauggugcauucgcuuu..........................................................    1          0           seq
    ..........................aggucuuccauggugcauucg..............................................................    2          0           seq
    .............................ucuuccauggugcauucgcuuu..........................................................    1          0           seq
    ..............................................................aaucauacaugguugaccuuu..........................    4          0           seq
    ..............................................................aaucauacaugguugaAcuuu..........................    2          1           seq
    ..............................................................aaucauacaugguugaccuuuu.........................    7          0           seq
    ..............................................................aaucauacaugguugaccuuuuu........................    25         0           seq
    ..............................................................aaucauacaugguugaccuuuuuu.......................    2          0           seq
    ...............................................................aucauacaugguugaccuuu..........................    3          0           seq
    ...............................................................aucauacaugguugaccuuuu.........................    51         0           seq
    ...............................................................aucauacaugguugaccuuuuu........................    6          0           seq
    ...............................................................aucauacaugguugaccuuuuuu.......................    2          0           seq
```
